# Artificial intelligence in polycystic ovarian syndrome management: past, present, and future

**DOI:** 10.1007/s11547-025-02032-9

**Published:** 2025-06-23

**Authors:** Jinyuan Wang, Ruxin Chen, Haojun Long, Junhui He, Masong Tang, Mingxuan Su, Renhe Deng, Yuru Chen, Rongqian Ni, Shuhua Zhao, Meng Rao, Huawei Wang, Li Tang

**Affiliations:** 1 Department of Reproduction and Genetics, The First Affiliated Hospital of Kunming Medical University, 295 Xichang Road, Kunming, 650032 Yunnan Province China; 2 Department of Gynecological Endocrinology, Jinan Maternity and Child Care Hospital Affiliated to Shandong First Medical University, Jinan, 250001 China; 3 Department of Dermatology, The Second Affiliated Hospital of Kunming Medical University, Kunming, 650101 Yunnan China; 4 Key Laboratory for Experimental Teratology of the Ministry of Education and Center for Experimental Nuclear Medicine, School of Basic Medical Sciences, Cheeloo College of Medicine, Shandong University, Jinan, 250012 Shandong China; 5 Department of Nuclear Medicine, Zhongshan Hospital, Fudan University, Shanghai, 200032 China; 6 Clinical Anatomy & Reproduetive Medieine Application Institute, Hengyang Medieal Sehool, University of South China, Hengyang, China; 7 Department of Plastic and Reconstructive Surgery, Third Xiangya Hospital, Central South University, Changsha, 410013 Hunan China

**Keywords:** Artificial intelligence, Polycystic ovary syndrome, Digital healthcare

## Abstract

**Background:**

Integrating artificial intelligence (AI) prospected in the practical clinical management of polycystic ovary syndrome (PCOS) promised significant improvement in efficiency, interpretability, and generalizability.

**Purpose:**

To delineate a comprehensive inventory of AI-driven interventions pertinent to PCOS across diverse clinical contexts.

**Evidence reviews:**

AI-based analytics profoundly transformed the management of PCOS, particularly in the domains of prediction, diagnosis, classification, and screening of potential complications.

**Results:**

Our analysis traced the principal applications of AI in PCOS management, focusing on prediction, diagnosis, classification, and screening. Furthermore, this study ventures into the potential of amalgamating and augmenting existing digital health technologies to forge an AI-augmented digital healthcare ecosystem encompassing the prevention and holistic management of PCOS. We also discuss strategic avenues that may facilitate the clinical translation of these innovative systems.

**Conclusion:**

This systematic review consolidated the latest advancements in AI-driven PCOS management encompassing prediction, diagnosis, classification, and screening of potential complications, developing a digital healthcare framework tailored to the practical clinical management of PCOS.

**Supplementary Information:**

The online version contains supplementary material available at 10.1007/s11547-025-02032-9.

## Introduction

Polycystic ovary syndrome (PCOS) is a common heritable, reproductive-metabolic endocrine disorder found in 10–15% of pre-menopausal women, characterized by sparse ovulation and hyperandrogenic anovulation [1]. Compelling evidence exists to implicate an increase in dyslipidemia, insulin resistance, glucose intolerance, endothelial dysfunction, inflammation, and oxidative stress as the primary pathological cause of PCOS [2]. Besides impacting the reproductive, PCOS is associated with an increased incidence of diabetes or obesity, high blood pressure, sleep apnea, depression and anxiety, and endometrial cancer [3–5]. With time and technological advances, the focus has shifted from developed countries to worldwide, to some primary defects of PCOS as a significant public health concern about female fertility in China [6]. Currently, according to two consecutive nationwide epidemiological surveys in reproductive-aged women in China, the prevalence of PCOS has increased by nearly 65% from a decade ago, and a weighted prevalence under the Rotterdam criteria reached 7.8% (95% CI 7.0%, 9.0%) in 2020 [7]. Therefore, in light of the declining female fertility and the accompanying huge burden on families and society, importance is attached to the effective assessment and management of PCOS with its underlying complications.

Medicine continues to endeavor for cost-effectiveness in the era of groundbreaking scientific developments in high-resolution and high-throughput technologies, making precision medicine that collects and analyzes vast, disparate datasets on individual health significant health breakthroughs. Recently, the dramatic development of artificial intelligence (Al) meets the high demands of mining and translating the huge amount of datasets, which entails streamlining the vast volumes of imaging and molecular testing into clinically actionable knowledge data, especially important for reproductive physicians in assisting the management of patients [8, 9]. In the field of digital health technologies, there has been rapid progress in the development of data science and AI-driven methodologies, stemming from the advent of powerful algorithms, exponential data growth, and upgraded computational power optimization in the medical and healthcare fields [10]. Numerous studies have validated that Al exhibited strong performance in addressing the obstacles of helping formulate better prevention strategies for high-risk populations and incorporation of patients-centered management for infertile patients who are unable to attend physician appointments in person, all of which deliver multitudes of real-time health information that might promote better self-management of patients, alleviating related substantial economic burden and workload on families and caregivers [11–13].

Nowadays, promising AI-based analytics, including machine learning (ML), neural networks, deep learning (DL), computer vision, and natural language processing, achieve a significant transformation for diagnoses and therapeutic monitoring of reproductive endocrinology in general, and specifically the screening and management of PCOS [14]. Introduced in 2023 by Su Z, the support vector machine (SVM) model developed on genetics data was noted to perform satisfactorily in exploring cuproptosis-related molecular clusters of PCOS, constructing the prediction model with an area under the curve (AUC) of 100% [15]. A combination of metabolomic signature (steroidome data of 15 steroids) and supervised ML provides a significant additive value (sensitivity and specificity of 100%) for response identification with PCOS from non-classic 21-hydroxylase deficiency [16]. Although there are many reasons to be hopeful for this application brought on by AI, plights remain for the successful utilization of AI in practical clinical frameworks. Based on the present background, we retrace the current major Al applications in PCOS and then illustrate the opportunities and challenges of building Al clinical translation. Moreover, we wish to meet the study's goal for the detailed investigation of the preferred cooperation between theory-based AI implementation and the prevention and management of real clinical PCOS which is a promising vision for the future of fertility preservation.

## Materials and methods

**Fig. 1:**
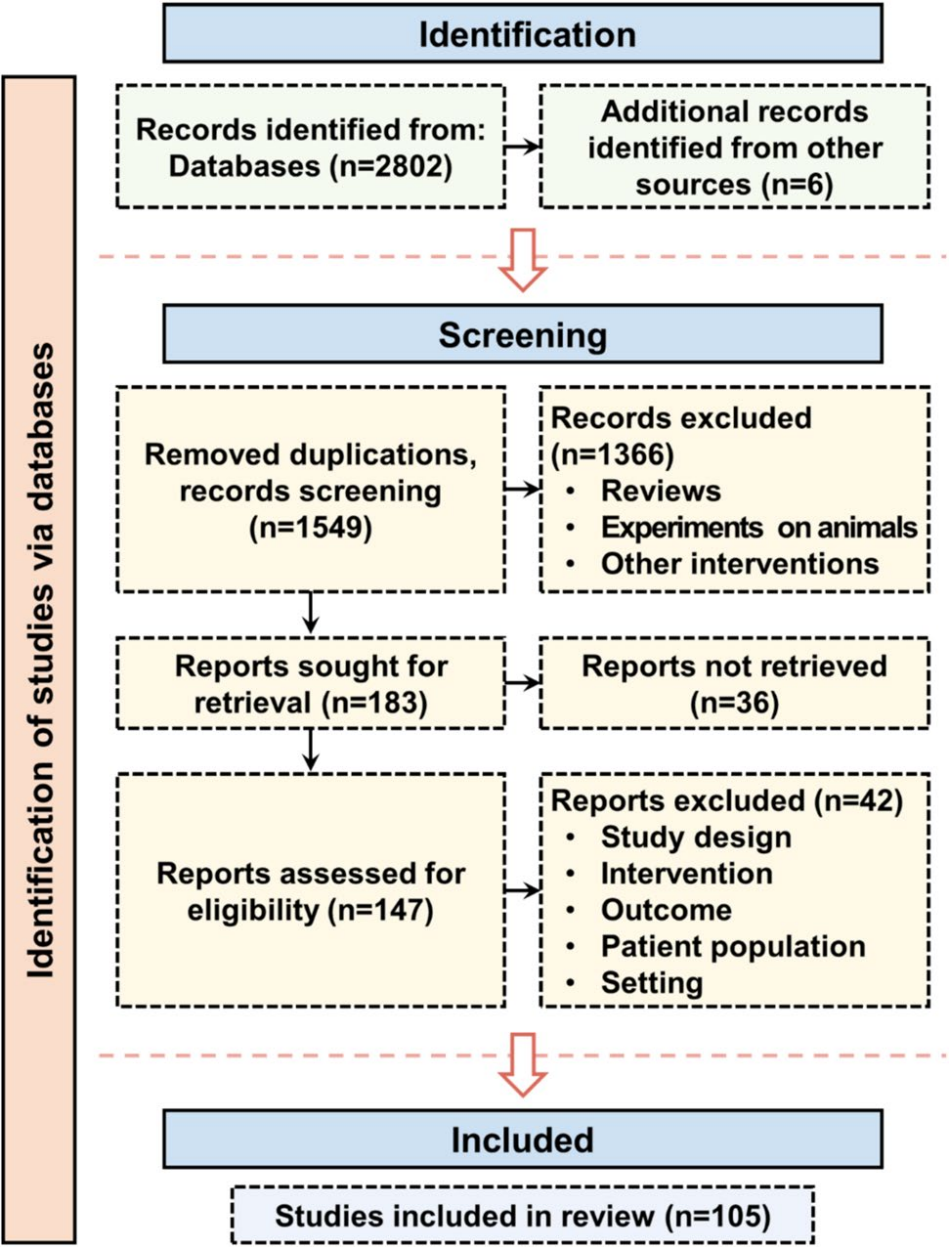
Selection process of the studies. Article selection flow chart for studies related to AI and PCOS according to Preferred Reporting Items for Systematic Reviews and Meta-Analyses (PRISMA) guidelines

We intend to investigate AI interventions in PCOS management, a search strategy followed by the ‘Preferred Reporting Items for Systematic Reviews and Meta-Analyses (PRISMA)’ statement applied in collaboration with PubMed, Embase, the Cochrane Central Register of Controlled Trials, the Web of Science, and Scopus Library, where the search included all articles from the time of inception of the dataset to Jan 2025 (Supplementary file 1) [17]. After three rounds of screening and eligibility assessing through Cohen's Kappa statistic (κ > 0.80), a total of 105 studies met our inclusion criteria, an outcome was included as a primary outcome only if clearly stated in the manuscript, and we did not contact authors to clarify this, among which are typical by a salient performance relative to standardized diagnostic criteria (such as the rates of AUC, sensitivity, specificity, and accuracy (Acc)), consistent with Barrera FJ, and Suha SA’s efforts (Fig. 1) [18, 19]. Collectively, the current research on AI applications for PCOS management is conducted in a high-quality qualitative avenue, which implies the validity of AI-based methodologies to manage PCOS. The general characteristics are summarized in Table 1.

**Table 1:** Major studies on the application of AI in PCOS

Studies	AI type	Data type	Task	Performance
*Objective: the prediction and prevention of risk factors for PCOS*				
Wang CY, 2025 [53]	RF, SGB, MARS, XGBoost, CatBoost	Information on demographics, biochemistry, and lifestyle	Forecasting PCOS and prioritizing the risk factors	AUC_ML_ = 66.69%, AUC_LR_ = 59.08%; Age, glutamic pyruvic transaminase, y-glutamyl transferase, triglyceride, white blood cell count, uric acid, and platelet were the hub risk factors
Ahmad R, 2024 [49]	CNN, LSTM, SMOTE	The dataset contains diagnostic and thorough information from the Kaggle repository	Predicting PCOS using cutting-edge DL methods	For CNN: Acc = 92.04%, ROC-AUC = 92.0%; For LSTM: Acc = 96.59%, ROC-AUC = 96.6%; For SOMTE: Acc = 94.31%, ROC-AUC = 94.3%;
Fu J, 2024 [62]	KNN, SVM, LR, RF, XGBoost	Clinical data from randomized controlled trial	Predicting the efficacy of metformin in improving insulin sensitivity among PCOS women with IR	For SVM: AUC 78.1%, Acc 70.1%, Spec = 70.8%, Sen = 70.8%, FI score = 0.706, outperforming all model; For KNN: FI score = 0.401; The SVM performed the best, whereas KNN performed the worst
Tan C, 2024 [46]	LR, NB, SVM	Genetics profiling downloaded from the GEO database	Predicting the risk of developing PCOS	For RF: cross-entropy = 11.1%, AUC = 96%; For NB: AUC = 99% (95% Cl: 0.968, 1.000) CXCR1, ACP5, CEACAM3, S1PR4, and TCF7 were hub genes
Vairachilai S, 2024 [51]	LR, SVM, KNN, DT, NB, RF, XGBoost, AdaBoost, CatBoost, blending	Information on age, body mass index, menstrual cycle length, follicle-stimulating hormone, and hair growth from Kaggle databases	Predicting PCOS based on demographic, clinical, and biochemical parameters	For traditional ML algorithms, Acc_LR_ = 91%, AUC_LR_ = 90%, outperforming all models For ensemble algorithms, Acc_Blending_ = 91%, AUC_Blending_ = 90%, PRE and REC = 88%, outperforming all models
Zad, Z, 2024 [52]	LR, SVM, RF, XGBoost	Clinical data, electronic health records	Predicting the risk of developing PCOS based on hormone levels, obesity, gravidity, and positive HCG	AUC_LR_ = 85%, AUC_SVM_ = 81%, AUC_XGBoost_ = 80%, AUC_FR_ = 82%
Zhang J, 2024 [63]	ARIMA, BAPC, HC	Data on PCOS incidence, prevalence, and years lived with disability from 1990 to 2019	Comprehensively assessing the global, regional, and national burden in PCOS incidence, prevalence, and years lived with disability in the next 20 years	The ARIMA and BAPC models showed a consistent increasing trend of the burden of PCOS
Alam Suha, S, 2023 [47]	Stacking ensemble ML classifiers	Clinical data, symptoms along with the PCOS diagnosis finding	Predicting the risk of developing PCOS	Acc = 95.7%, PRE = 95.2%, REC = 95.2%, and F1 score = 0.95
Su Z, 2023 [15]	RF, SVM, GLM, XGBoost	Genetics profiling downloaded from the GEO database	Exploring PCOS-specific molecular clusters related to cuproptosis	AUC_RF_ = 90.5%; AUC_XGBoost_ = 90.5%; AUC_GLM_ = 78.6%; AUC_SVM_ = 100%, outperforming all models
Wang W, 2023 [60]	XGB, SVM, MLP	Clinical data, risk factors	Exploring PCOS-specific prediction models related to tongue and pulse	For training set: AUC = 0.979 ± 0.003, Acc = 0.957 ± 0.003, Sen = 0.979 ± 0.014, Spec = 0.933 ± 0.013; For validation set: AUC = 0.980 ± 0.016, Acc = 0.944 ± 0.015, Sen = 0.971 ± 0.019, Spec = 0.956 ± 0.024
Zhan W, 2023 [55]	Multiple LR, XGBoost	Clinical data quantified seven bisphenol analogs in urine samples	Exploring PCOS-specific prediction models related to endocrine-disrupting chemicals (bisphenol analogs)	Mixed exposure to seven bisphenol analogs was found to be positively associated with the odds of PCOS Women who were overweight or obese tended to have a stronger association between bisphenol analogs and PCOS than normal-weight women
Zigarelli A, 2023 [48]	CatBoost model, *k*-means clustering algorithm, *K*-fold cross-validation	Clinical data, risk factors	Developing self-diagnostic prediction models for PCOS	For the patient models, Acc = 81–81.5%; For the provider models, Acc = 87.5–90.1%
Garzia E, 2022 [61]	ANN	Clinical data, clinical features, and treatment outcomes	Identifying reliable predictors of response to metformin therapy for weight loss and reduction in plasma androgen levels of PCOS	Menstrual pattern imbalance and ovarian androgen excess are the best predictors of metformin response in PCOS Baseline plasma testosterone level is a sensitive marker to predict treatment compliance
Wang DD, 2022 [21]	The maximal effect model	Clinical data, risk factors	Exploring the effect of carnitine supplementation on body weight in PCOS and predicting an appropriate dosage schedule	The treatment times to achieve 25%, 50%, 75%, and 80% (plateau) Emax of carnitine supplementation on body weight are 1.2, 3.6, 10.8, and 14.4 weeks, respectively The optimal therapeutic effect of carnitine supplementation on body weight in PCOS is 250 mg/day for at least 14.4 weeks
Lv W, 2021 [59]	MIL, MLP, CBAM, U-Net, ResNet	Images, the full-eye images	Estimating the potential of scleral changes in PCOS	AUC = 0.979 ± 0.003, Acc = 0.929 ± 0.007, PRE = 0.940 ± 0.006, REC = 0.928 ± 0.006, FI score = 0.934 ± 0.006
Ho CH, 2020 [44]	SVM, RF, GMM	Genetics profiling downloaded from the NCBI and GEO database	Identifying the hub genes in PCOS	For fivefold cross-validation models: Acc = 100%, AUC = 100%, Sen = 100%, Spec = 100%; for threefold cross-validation models: Acc = 100%, AUC = 100%, Sen = 100%, Spec = 100%; for twofold cross-validation models: Acc = 99.65%, AUC = 99.23%, Sen = 98.52%, Spec = 100%; SVM with 5 and threefold cross-validation Transporter activity, catalytic activity, tyrosine phosphorylation of STAT5 protein, and immune response may participate in the pathogenesis of PCOS
Rodriguez EM, 2020 [54]	Bayesian network	Virtually generated, clinical data signs and symptoms	Estimating if the irregular cycle feature generates PCOS	The creation of the irregular cycle feature may reduce the time to PCOS diagnosis and facilitate conversations between users and physicians through the results screens and doctor's report
Zhang XZ, 2018 [45]	SVM, DT, KNN	Genetics, genome	Identifying the hub genes in PCOS	For SVM: PRE = 81%, REC = 71%, F1 score = 0.75, AUC = 80%, outperforming all other model IGF1, IGF2, and SMAD3 were hub genes
Coskun S, 2013 [42]	HC	Genetics profiling of granulosa cells in FF	Evaluating the dose-dependent differences in gene expression of granulosa cells following various doses of HCG treatment	10,000 IU HCG resulted in minimal changes in the gene expression profiles of granulosa cells as compared with 5000 IU
Zhou S, 2013 [43]	RF, SVM	The RNA sequencing data	Assessing the relationship between m6A regulators and the occurrence risk of PCOS	For RF: Spec = 100%, outperforming all other models YTHDF1, RBM15, and METTL14 were optimal m6A regulators related to the occurrence risk of PCOS
Zhao SY, 2005 [41]	SVM	Serum proteomic profiling	Identifying the hub proteins of PCOS	Sen = 86.7%, Spec = 83.3%, PPV = 87.2%

*Objective: the diagnosis and distinction of PCOS*				
Chen JY, 2025 [85]	EL, RF, LR, SVM, OPLS-DA, PCA	Metabonomic profiling, lipid metabolites	Identifying serum biomarkers in PCOS patients	For the constructed diagnostic panel model: AUC = 81.5%, Acc = 74%, Spec = 88%, Sen = 70% PI (18:0/20:3)-H and PE (18:1p/22:6)-H was hub biomarkers
Chen W, 2025 [76]	SVM, CIBERSORT, LASSO, XGBoost	RNA-seq analysis on granulosa cells	Exploring immune cell infiltration patterns in PCOS	AUC_SVM_ = 79.5%, AUC_XGBoost_ = 87.5%; CNTN2, CASR, CACNB3, and MFAP2 were hub biomarkers
Wang M, 2025 [82]	LDA, C5, NB, NNET, SVM, RF, KNN, LogitBoost, VIP	Metabonomic profiling, steroidome data	Distinguishing PCOS based on the steroid hormone biosynthesis pathway	For RF: Acc = 93.8%, AUC = 98.9%, kappa ratio = 90.6%, logLoss = 20%, outperforming all models
Yang R, 2025 [97]	SSFSE, DL	Imaging, high-resolution ovarian MRI	Distinguishing PCOS based on the follicle count	DL reconstruction high-resolution SSFSE imaging is a more dependable method for identifying polycystic ovaries
Moral, P. 2024 [93]	NB, KNN, RF, AdaBoost, CystNet	Imaging, ultrasound images from the Kaggle website	Developing new diagnostic models for PCOS detection using multilevel thresholding of ultrasound images	With a fivefold cross-validation process, for the dense layer approach: Acc = 96.54%, PRE = 94.21%, REC = 97.44%, Spec = 95.92%; For the RF classifier Acc = 97.75%, PRE = 96.23%, REC = 98.29%, and Spec = 97.19%
Kermanshahchi J, 2024 [94]	CNN	Imaging, pelvic ultrasound images from Kaggle	Accurately identifying PCOS pelvic ultrasound images	PRE = 82.6%, REC = 100%, Spec = 100%, Sen = 100%, Acc = 100%, F1 score = 0.905
Khushal R, 2024 [98]	GNB, SVM, AdaBoost	Hormonal imbalance dataset from the Kaggle website	Developing a novel fuzzy ML logic utilization for PCOS diagnostic models based on a hormonal imbalance dataset	For the PCOS dataset, the adaptive fuzzy ML model outperformed all models; For the diabetes dataset (validation dataset), SVM outperformed all models
Liu X, 2024 [71]	RF	Genetics profiling downloaded from the GEO database	Developing new diagnostic models for PCOS	ACSL5, NLRP12, CCRL2, and CEACAM3 were hub genes
Paramasivam GB, 2024 [90]	SD_CNN, SVM, RF, LR	Imaging, ovarian ultrasound images	Detecting the condition and treatment of PCOS	AUC_SVM_ = 96.43%; AUC_LR_ = 96.43%;
Przewocki J, 2024 [79]	RF	Proteomic profiling	Exploring new diagnostic models for PCOS based on the proteomic composition of FF	APOA1 and A2M were the hub proteins involved in high-density lipoprotein assembly Myosin light polypeptide 6 was the hub protein
Qu Y, 2024 [83]	NN, GB, LR, RF, SGD	Metabonomic profiling, urine metabolite fingerprints in patients with different gynecological diseases	Identifying various gynecological diseases that may nourish infertility	For the training set: AUC > 93%; For testing sets: AUC > 85% For NN: AUC_training sets_ = 99.6%, AUC_new dataset_ = 98%, outperforming all models
Shanmugavadivel K, 2024 [95]	LR, NB, SVM, CNN, VGG16	Clinical data, clinical features dataset; ultrasound imaging dataset from Kaggle	Developing new diagnostic models for PCOS	For clinical features dataset: Acc_SVM_ = 94.44%, outperforming all models For ultrasound imaging dataset: Acc_VGG16_ = 98.29%, outperforming all models
Shen HH, 2024 [72]	LASSO, RF, SVM-RFE	Genetics profiling downloaded from the GEO database	Developing new diagnostic models for PCOS	ACSS2, LPIN1, and NR4A1 were hub genes. AUC_ACSS2_ = 96%, AUC_LPIN1_ = 90%, AUC_NR4A1_ = 76%
Silva EL, 2024 [102]	LR	Clinical data, electronic health records	Identifying individual-level and spatial predictors of missed diagnosis of PCOS	In multivariable models, Black/African American patients, relying on Medicaid or charity for insurance were more likely to have missed a PCOS diagnosis In univariate models, higher social vulnerability index scores were associated with increased odds of missed diagnosis
S S, 2024 [96]	RF, k-star, SGD, FCM, F-Net	Dataset 1: abdominal ultrasound images; Dataset 2: ultrasound images	Developing new diagnostic models for PCOS	Acc_Dataset 1_ = 95%, Acc_Dataset 2_ = 97.5%
Wang F, 2024 [99]	AdaBoost, XGBoost, DT, KNN, LR, RF	Clinical data, serum OVGP1 concentrations, hormones, and blood biomarkers	Investigate the diagnostic value of OVGP1 levels for PCOS	OVGP1 was hub gene For the training set: AUC_XGBoost_ = 95.3% (95% CI = 0.916, 0.990); For the testing set: AUC_XGBoost_ = 90.7% (95% CI = 0.855, 0.959)
Wang J, 2024 [73]	LASSO, SVM-RFE	downloaded from the GEO database	Identifying acetylation-related genetic markers associated with PCOS	SGF29, NOL6, KLF15, and INO80D were hub genes
Xuan Y, 2024 [81]	PCA, OPLS-DA	Metabonomic profiling, vaginal metabolites	Identifying the metabolic characteristics and potential biomarkers of PCOS in Chinese women of reproductive age	Most differential metabolites were enriched in pathways associated with linoleic acid metabolism, phenylalanine metabolism, tyrosine metabolism, nicotinate and nicotinamide metabolism or arachidonic acid metabolism
Yang Y, 2024 [74]	LASSO, RF, SVM-RFE, XGBoost	Genetics profiling downloaded from the GEO database	Exploring PCOS-specific biomarkers related to mitophagy	MAP1LC3A was hub gene, AUC > 0.8. XGBoost outperformed all models
Yang Z, 2024 [75]	LASSO, RF, SVM-RFE	Transcriptome sequencing on granulosa cells	Exploring PCOS-specific biomarkers	CCR7 was hub gene
Yu J, 2024 [84]	RF	High-performance liquid chromatography-tandem spectrometry	Distinguishing PCOS from metabolic composition of FF	AUC = 80.5%; Differential metabolites primarily participated in the metabolism of glycerophospholipids and the biosynthesis of steroid hormones The concentrations of prostaglandin E2 in the FF of PCOS were negatively associated with the proportion of high quality
Bachelot G, 2023 [16]	OPLS-DA	Metabonomic profiling, steroidome data (set of 15 steroids)	Distinguishing PCOS from non-classic 21-hydroxylase deficiency without adrenocorticotrophic hormone testing	Sen = 100%, Spec = 100%
Elmannai H, 2023 [100]	LR, RF, DT, NB, SVM, KNN, XGBoost, AdaBoost	Clinical data, PCOS dataset from Kaggle	Providing model explanations to ensure efficiency, effectiveness, and trust of AI model for PCOS diagnosis	Bayesian optimization is used to optimize ML models Acc = 98.87%, PRE = 98%, REC = 98. 87%, and F1 score = 0.9889
Emanuel RHK, 2023 [101]	CNN	A random subset of 5000 posts from the PCOS subreddit with laboratory test results	Presents AI systems for PCOS screening	Acc = 98%
Wu Y, 2023 [70]	ANN, RF	Genetics profiling downloaded from the GEO database	Developing new diagnostic models for PCOS	For the training set: AUC = 96.5%; For the validation set: AUC = 82.9%
Xu WL, 2023 [86]	PCA, OPLS-DA, PLS-DA	Metabonomic profiling, the metabolite fingerprints of serum and FF samples in PCOS patients	Distinguishing PCOS from metabolic composition of FF and serum	Three discriminated metabolites (1-methylhistidine, threonine, and citrate) in both serum and FF were altered in PCOS patients Abnormal energy metabolism, lipid metabolism and amino acid metabolism were detected in PCOS patients
Yang R, 2023 [35]	DL-reconstructed SSFSE	Imaging, high-resolution ovarian MRI	Investigating the diagnostic performance of AI on follicle counting	SSFSE-DL images showed less blurring artifact, subjective noise, and better clarity of the follicles
Yu J, 2023 [87]	OPLS-DA, XGBoost	Metabonomic profiling, the metabolite fingerprints of serum bile acid profiles	Identifying potential biomarkers for PCOS pathogenesis	CDCA and LCA combined with testosterone were hub biomarkers
Na Z, 2022 [67]	SVM-RFE, LASSO	Genetics profiling downloaded from the GEO database PCOS-related biomarkers	Screening potential biomarkers of PCOS	HDDC3 and SDC2 were hub biomarkers
Qu J, 2022 [68]	RF, SVM, GLM	Genetics profiling downloaded from the GEO database	Identifying several immune-related biomarkers and constructing an anemogram model for the diagnosis of PCOS	AUC > 70%
Suha SA, 2022 [25]	CNN, DNN	Imaging, ovary ultrasonography scans	Investigating the diagnostic performance of AI on ovary ultrasonography scans	Acc = 97.8%
Wang W, 2022 [78]	HC	Proteomic profiling based on mass spectrometry of FF	Identifying the potential differentially expressed proteins of PCOS	Inflammatory response, complement and coagulation cascades, activation of the immune response, lipid transport, and regulation of protein metabolic process were hub pathogenesis of PCOS
Zhang N, 2021 [20]	CNN, GCN, RF, NB, SVM, DeepGP	Curated disease gene, causative loci	Prioritizing susceptible genes of PCOS	For DeepGP: Acc = 84.5%, AUPR = 83.3%, outperforming all models
Zhang X, 2021 [92]	KNN, RF, XGBoost, stacking classification model	Clinical data, metabolic data	Screening the metabolic profile changes of PCOS	For KNN: Acc = 89.32%, Sen = 87%, Spec = 90%, outperforming all models
Xie NN, 2020 [69]	RF, ANN	Genetics profiling, microarray	Identifying gene biomarkers and constructing a diagnostic model for PCOS	For ANN: Acc = 73%, Sen = 73%, Spec = 75%, outperforming all models
Castro V, 2015 [103]	Algorithm using natural language processing and codified data	Clinical data, electronic medical data	Developing the diagnostic performance of AI in identifying PCOS patients	PPV = 68%
Liu S, 2015 [77]	HC	Small RNA deep sequencing	Identifying miRNA expression patterns of cumulus cells from PCOS patients	Hub miRNAs of PCOS regulated genes with Wnt- and MAPK signaling pathways, oocyte meiosis, progesterone-mediated oocyte maturation, and cell cycle functions
Haoula Z, 2014 [88]	LASSO regression, OPLS-DA	Plasma lipidomic profiles	Identifying lipid biomarkers of PCOS at different stages of menstrual cycle	For PCOS vs luteal: Spec_OPLS-DA_ = 85%, Sen_OPLS-DA_ = 95%; Spec_LASSO_ = 85%, Sen_LASSO_ = 95%; For PCOS vs follicular: Spec_OPLS-DA_ = 55%, Sen_OPLS-DA_ = 75%; Spec_LASSO_ = 70%, Sen_LASSO_ = 50%; For PCOS vs mid-cycle: Spec_OPLS-DA_ = 45%, Sen_OPLS-DA_ = 65%; Spec_LASSO_ = 55%, Sen_LASSO_ = 40%;
Deng Y, 2011 [91]	Watershed object growing algorithm, level set method, boundary	Imaging, ultrasound images	Automating diagnoses of PCOS based on ultrasound images	RR = 89.4%, MR = 7.45%
Borro M, 2007 [80]	HC	Proteomics profiling, serum proteins of T cells	Investigating the susceptible T cell protein of PCOS	The Rho GDP-dissociation inhibitor 1, the F-actin capping protein α-1 subunit, cofilin-1, cathepsin D, 3-hydroxybutyrate dehydrogenase, protein disulfide isomerase A3, RKIP, peroxiredoxin-1, and α-enolase were down-expressed in PCOS, the platelet basic protein and superoxide dismutase were up-regulated
Matharoo-Ball B, 2007 [66]	ANN	Proteomics profiling, serum proteins/peptide biome	Investigating the susceptible protein/peptide of PCOS	For ANN: Acc = 100%, AUC > 80%, outperforming all models
Lehtinen JC, 1997 [27]	TPFFN and SOM	Clinical data, hormones, and blood biomarkers	Presenting AI systems for 2-dimensional visualization of PCOS	For fuzzy TPFFN: Acc = 97%, outperforming all models

*Objective: the classification and evaluation of PCOS*				
Guo B, 2024 [108]	LR, RF	Clinical data, clinical features dataset	Characterizing the PCOS patients with and without IR	For the training, internal validation, and external validation sets, the AUC_training_ = 91.1% (95% Cl, 0.878–0.911), AUC_Internal validation_ = 84.2% (95% Cl, 0.771–0.842), AUC_External validation_ = 90.1% (95% Cl, 0.856–0.901)
Lim J, 2024 [117]	LR, ANN, XGBoost, SVM	Clinical data, exhibiting one or more of the five TCM patterns	Developing an effective predictive model for classifying TCM patterns in PCOS	For XGBoost: Acc = 78.7%, F1 score = 0.9519, hamming loss = 0.0481 with RFECV-optimized features, outperforming all models
Liu W, 2024 [107]	Apriori rules algorithm, PCA	Genetics profiling downloaded from the GEO database; Microarray analysis on granulosa cells from PCOS patients	Indicating PCOS functional and classification markers based on NA and HA Constructing knowledge graphs and predicting drugs for NA PCOS and HA PCOS based on transcriptome data	For hub genes: NA PCOS were IL6R and CD274, HA PCOS was CASR For specific causes of female infertility: HA PCOS included hyperandrogenism, cholesterol, and adiponectin; NA PCOS included immunity terms and hyperinsulinism; For potential drug: HA PCOS might be promoted or inhibited by androgen, tamoxifen, and flutamide; NA PCOS might be promoted or inhibited by human albumin, herapin, insulin, adenosine, liothyronine sodium, and antibiotics
van der Ham K, 2024 [115]	HC	Clinical data, clinical features dataset	Identifying distinct PCOS subtypes, and assessing additional clinical variables	Metabolic (41%), reproductive (18%), and background (41%) PCOS subtypes were identified
Lim J, 2023 [116]	KNN, SVM, DT, RF, LR, voting, LSTM, k-fold cross-validations training	Clinical data, the pulse-wave parameters	Classifying PCOS based on radial pulse-wave analysis	Voting and LSTM are outperforming all models. They both attained a testing AUC = 72.174% and F1 score = 0.818; AUC_Voting_ = 71.5%, AUC_LSTM_ = 72.2%
Fruh V, 2022 [113]	Rule-based classifier model	Clinical data, radiologic report data	Classifying PCOM by ultrasound	Acc = 97.6% (95% CI 96.5, 98.5%)
Kangasniemi MH, 2022 [24]	Al model training, based on the supervised training of CNN	Imaging scanned whole slide images	Discerning quantitative differences in endometrial immune cells between cycle phases and between PCOS and non-PCOS	AI is objective and can efficiently analyze endometrial compartments separately
Silva IS, 2022 [22]	BorutaShap method, RF	Clinical data, risk factors	Stratifying patients into different phenotypic clusters	Acc = 86.2%
Fulghesu AM, 2021 [111]	OPLS-DA, NMR	Urine metabolomics	Identifying a panel of urinary biomarkers of hyperinsulinemia and IR in PCOS	The OPLS model indicated that the urine NMR profile had a good fit and prediction ability for the AUC OGTT, with R2 = 0.813
Dapas M, 2020 [29]	SVM, RF, GMM, HC	Genome-wide association, biochemical and genotype	Identifying the original subtype clusters of PCOS	The RF classifier yielded the lowest mean subtype misclassification rate (13.2%)
Cheng JJ, 2019 [112]	Text-based ML algorithms	Clinical data, pelvic ultrasound reports	Classifying of PCOM in pelvic ultrasounds	Task 1 Acc = 97.6% (95% Cl 96.5, 98.5%) Task 2 Acc = 96.1% (95% Cl 94.7, 97.2%)
Kumar HP, 2014 [114]	PNN, SVM, RBF	Imaging, ultrasound	Automated classifying of the ovaries based on the biomarking	Acc_PNN_ = 98%, outperforming all models
Zhang X, 2014 [110]	OPLS-DA, VIP, S-plot	The plasma phospholipid fatty acid profile	Characterizing the PCOS patients with and without IR	Nervonic acid (C24:1 n-9) and dihomo-y-linolenic acid (C20:3 n-6) were hub potential fatty acid biomarkers of PCOS and its IR complication
Zhao S, 2007 [109]	SVM	Metabonomic profiling, serum protein peak spectrum	Screening out marker proteins from differentially expressed proteins and establishes three diagnostic models for PCOS	For IR: Acc = 83.33%, Sen = 80%, Spec = 86.67%, PPV = 84.62%, Kappa value = 75%; For non-IR: Acc = 88.33%, Sen = 86.67%, Spec = 90%, PPV = 88.33%, Kappa value = 83.3%

*Objective: screening complications and educating patients*				
Cai J, 2024 [146]	LR, linear regression, HC	Clinical data, anthropometric measurements, glucose-lipid profiles, reproductive hormones, and body composition	Identifying if the association of body fat mass and skeletal muscle mass with cardiometabolic risk differed in PCOS subtypes	Body fat mass and skeletal muscle mass were synergistically associated with higher cardiometabolic risk in PCOS women
Devranoglu B, 2024 [152]	ChatGPT-4	Queries spanned diagnostic standards, therapeutic approaches, monitoring procedures for PCOS	Assessing the efficacy of ChatGPT-4 in delivering precise and comprehensive answers to inquiries regarding managing	For true/false questions: Acc_initial_ = 100%, Acc_30-day_ = 100%; For the open-ended category, Acc_initial_ = 5.53 ± 0.89, Acc_30-day_ = 5.88 ± 0.43; For open-ended queries: Acc_initial_ = 2.35 ± 0.58, Acc_30-day_ = 2.92 ± 0.29; For the multiple-choice category: Acc_initial_ = 5.96 ± 0.44, Acc_30-day_ = 5.92 ± 0.6; For completeness scores for multiple-choice questions: Acc_initial_ = 2.98 ± 0.18, Acc_30-day_ = 2.97 ± 0.25
Gunesli I, 2024 [151]	ChatGPT-4, ChatGPT-3.5 and Gemini	Questions for PCOS based on current clinical guideline	Comparing the responses of ChatGPT-4, ChatGPT-3.5, and Gemini to PCOS-related questions using the latest guideline	Acc_ChatGPT-4_ = 68.2%, and Acc_ChatGPT-3.5_ = 56.8%, outperforming all models, while Acc_Gemini_ = 29.6%; FRE_Gemini_ = 36.7 ± 10.2, outperforming all models Tendency to Hallucinate scores were observed as 3.5 ± 1, 3.8 ± 1, and 3.1 ± 1.1 for ChatGPT-4, ChatGPT-3.5, and Gemini
Ge Y, 2024 [131]	The topological matrix	Genetics profiling downloaded from the GEO database	Identifying PCOS-related genes in endometrial cancer	Genes related to the intracellular aromatic compound metabolic pathway can be used to immunize endometrial cancer
He J, 2024 [124]	RF, SVM-RFE, LASSO	Genetics profiling downloaded from the GEO database	Identifying PCOS-related genes of recurrent spontaneous abortion	FAM166B was the hub gene
Huang J, 2024 [128]	RF	Genetics profiling downloaded from the GEO database	Identifying PCOS-related genes of damaged oocyte quality based on ferroptosis in granule cells	Dysregulation of ferroptosis-related genes (ATF3, BNIP3, DDIT4, LPIN1, NOS2, NQO1, SLC2A1, and SLC2A6) is associated with impaired oocyte quality in PCOS granulosa cells
Huang X, 2024 [144]	MLP, RF	Clinical data, clinical features dataset	Identifying inflammatory factors in FF that may affect the embryonic development of PCOS; Assessing the anxiety and depression tendencies of PCOS patients; Developing an Al model to predict pregnancy outcomes	The Acc of the RF model in predicting pregnancy outcomes in patients with or without PCOS was 95.6% and 91.1%
Lee S, 2024 [137]	CNN	Clinical data, clinical features dataset	Identifying PCOS-related genes of recurrent implantation failure based on endometrial histology features and calculating the areas occupied by epithelial and stromal cells	For delineating epithelial: Acc = 92.40%, F1 score > 0.82; For stromal compartments: Acc = 99.23%, F1 score > 0.96
Lee S, 2024 [137]	CNN	The CD138 + cell percentages based on cycle phases, ovulation status, and endometrial receptivity	Identifying endometrial CD138 + plasma cells in infertility-related PCOS	Acc between the Al and human evaluation methods (intraclass correlation; 0.76, 95% CI 0.36–0.93)
Luo Y, 2024 [133]	LASSO, RF, SVM-RFE	Genetics profiling downloaded from the GEO database	Identifying PCOS-related genes of atherosclerosis	AUC = 79.8%; DAPK1 was hub protein
Liang H, 2024 [134]	LASSO, SVM-RFE	Genetics profiling downloaded from the GEO database and GeneCards database	Identifying PCOS-related genes of MDD	The mitochondrial functional gene NPAS2 was the hub gene
Mogos R, 2024 [125]	DT, NB, RF, SVM	Clinical data, clinical features dataset	Predicting adverse obstetrical outcomes in PCOS	For gestational diabetes: AUC_RF_ = 78.2%; For fetal macrosomia: AUC_FR_ = 89.7% For preterm birth: AUC = 90.1%, outstanding all models
Naroji S, 2024 [149]	NMF, LDA, LR	PCOS-related posts on TikTok, Instagram, and Reddit	Assessing the extent, content, and engagement of PCOS-related information across social media platforms	For coherent topics of PCOS: TikTok and Instagram were"Weight"and"Diet", and interactions with medical providers were discussed in 30% of posts including symptoms, interventions, interactions with the medical system, and information-seeking 45% of TikTok posts and 89% of Instagram posts presented a financial conflict of interest The most comments in Reddit posts were"Symptom Management"
Tharayil SP, 2024 [141]	HC	Clinical data, anthropometric parameters, and detailed personal and family history	Indicating a link between mitochondrial dysfunction and central obesity in PCOS	Mitochondrial DNA copy number was negatively correlated with the waist-to-hip ratio, triglycerides, and positively related to high-density lipoprotein cholesterol
Ulug E, 2024 [150]	ChatGPT-4	Answers about PCOS-related general nutritional recommendations and weight management in English and Turkish formed by ChatGPT-4	Evaluating the reliability, quality, and readability of ChatGPT's responses to PCOS-related nutritional recommendations	For the mean modified DISCERN scores: English versions = 27.6 ± 0.87, Turkish versions = 27.2 ± 0.87; For the global quality score: English versions = 100%, Turkish versions = 90.9%
Wu Y, 2024 [127]	KM, SVM, RF, GLM, LASSO, XGBoost	Proteomic profiling	Identifying PCOS-related proteins of infertility	TIA1 and COL5A1 were hub proteins
Zhang X, 2024 [130]	LASSO, SVM-RFE	Genetics profiling downloaded from the GEO database	Identifying the PCOS-related genes associated with ferroptosis	ENPP2 was the hub gene in the progression from PCOS to endometrial carcinoma
Zhang W, 2024 [132]	LASSO, RF, SVM-RFE	Genetics profiling downloaded from the GEO database	Identifying PCOS-related genes in atherosclerosis	AUC > 90%; MMP9 and P2RY13 were hub genes of atherosclerosis in PCOS
Chakraborty P, 2023 [140]	Dynamic Bayesian network	Clinical data, hormonal and biochemical data	Identifying the PCOS-related risk of spontaneous miscarriage based on hyperhomocysteinemia	AUC = 77.8%, Sen = 80.7%, Spec = 70.6%
Chen W, 2023 [123]	LASSO, SVM-RFE and RF	Genetics profiling downloaded from the GEO database	Identifying PCOS-related genes of recurrent implantation failure	GLIPR1 and MAMLD1 were hub genes of recurrent implantation failure in PCOS
Deshmukh H, 2023 [28]	Unsupervised ML algorithm (k-means clustering)	Clinical data and a replication cohort (Hull PCOS Biobank)	Determining if a subset of women with PCOS had higher androgen levels predisposing them to metabolic syndrome	Identifies PCOS with significantly higher luteinizing hormone, free androgen index, and androstenedione levels are associated with a higher metabolic syndrome score
Dasgupta S, 2023 [145]	RF	Clinical data in a randomized controlled trial	Comparing the prevalence of ultra-low dose hormonal pill-triggered atherosclerotic cardiovascular disease in non-obese PCOS women	Non-obese PCOS women on ultra-low-dose pill have a lower risk of acquiring future atherosclerotic cardiovascular disease
S NLC, 2023 [142]	EDA, elbow clustering algorithms	Clinical data, clinical features dataset	Predicting the risk of developing diabetes in PCOS	Diabetes recorded a positive correlation of 95% to PCOS
Ozer G, 2023 [136]	LR, RF	Clinical data, risk factors	Predicting the risk of PCOS that causes first-trimester pregnancy loss in good-quality FET cycles	Sen = 69%, Spec = 68%, AUC = 67.6%, overall success rate = 60.8%
Amini P, 2021 [139]	SVM, XGBoost, LR, RF, NB, LDA	Clinical data, the patient's demographic and clinical variables for 6071 cycles from March 21, 2011 to March 20, 2014	Classifying successful deliveries after IVF according to couples'characteristics and available data on oocytes, sperm, and embryos	For exposed RF: Acc = 81%, outstanding all models For the importance of variables, MDA_total number of embryos_ = 12.94, MDA_number of injected oocytes_ = 10.9, MDA_cause of infertility_ = 8.2, MDA_female age_ = 6.73, MDA _PCOS_ = 6.13, outstanding all predicting factors
Huang X, 2021 [126]	ANN	Metabonomic profiling, Raman spectra of FF	Evaluating PCOS predictive value for oocyte development potential and clinical pregnancy	For high-quality blastocysts: F1 score = 0.902; For low-quality blastocysts: F1 score = 0.898; For classifying pregnancy success spectra: F1 score = 0.7937; For pregnancy failure spectra: F1 score = 0.6486; For the blastocyst developmental ANN model: AUCs = 0.89 ± 0.039, Sen = 0.692 ± 0.037, Spec = 0.948 ± 0.031; For the ANN model predicting the IVF outcome: AUCs = 0.72 ± 0.023, Sen = 0.738 ± 0.049 and Spec = 0.63 ± 0.043
Kodipalli A, 2021 [143]	DT classifier, KNN, SVM, fuzzy TOPSIS	The local-specific dataset collected on a spectrum of women	Estimating the likelihood of having PCOS and associated mental health issues	For fuzzy TOPSIS: Acc = 98.20%, outperforming all models
Arffman R, 2019 [122]	PCA, OPLS-DA, HC, SOM	Proteomic profiling, the plasma proteome	Identifying PCOS-related proteins of infertility	Properdin and IGF2 were hub proteins, and properdin reached the best predictive Acc for PCOS (AUC = 1) abundances correlated with AMH levels in pregnant women

*Acc* accuracy; *AdaBoost* adaptive boosting; *AI* artificial intelligence; *AMH* anti-Mullerian hormone; *ANN* artificial neural networks; *APOA1* apolipoprotein-A1; *ARIMA* autoregressive integrated moving average; *AUPR* area under the precision–recall curve; *A2M* alpha-2-macroglobulin; *BAPC* Bayesian age–period–cohort; *ChatGPT* chat generative pretrained transformer; *CIBERSORT* cell-type identification by estimating relative subsets of RNA transcripts; *CNN* convolutional neural network; *C5* C5.0 algorithm; *DL* deep learning; *DNN* deep neural network; *DT* decision tree; *EL* ensemble learning; *FCM* fuzzy C-means; *FET* frozen-thawed embryo transfer; *FF* follicular fluid; *F-Net* follicles net; *FRE* Flesch reading ease; *LASSO* least absolute shrinkage and selection operator; *GB* gradient boosting; *GCN* graph convolutional network; *GDP* guanosine diphosphate; *GEO* gene expression omnibus; *GLM* generalized linear model; *GMM* Gaussian mixed model; *GNB* Gaussian naive Bayes; *HA* hyperandrogenemia; *HC* hierarchical clustering; *HCG* human chorionic gonadotropin; *HDDC3* HD domain containing 3; *IGF2* insulin-like growth factor II; *IR* insulin resistance; *IU* international unit; *IVF* in vitro fertilization; *KNN* k-nearest neighbor; *LASSO* least absolute shrinkage and selection operator; *LDA* linear discriminant analysis; *LogitBoost* boosted logistic regression; *LR* logistic regression; *LSTM* long short term memory networks; *MARS* multivariate adaptive regression splines; *MDA* mean decrease in accuracy; *MDD* major depressive disorder; *MIL* multi-instance learning; *ML* machine learning models; *MLP* multilayer perceptron; *MR* misidentification rate; *MRI* magnetic resonance imaging; *NA* non-hyperandrogenemia; *NB* naïve Bayes classifier; *NCBI* national center for biotechnology information; *NMR* nuclear magnetic resonance; *NNET* neural network; *NMF* non-negative matrix factorization; *OPLS-DA* orthogonal partial least squares discriminant analysis; *OSSM* optimized salp swarm; *PCA* principal component analysis; *PCOM* polycystic ovary morphology; *PCOS* polycystic ovary syndrome; *PE* phosphatidylethanolamine; *PI* phosphatidylinositol; *PNN* probabilistic neural network; *PPV* positive predictive value; *PRE* precision; *RBF* radial basis function; *REC* recall; *RF* random forest; *RFECV* recursive feature elimination with cross-validation; *RKIP* Raf kinase inhibitor protein; *ROC-AUC* area under the receiver operating characteristic curve; *RR* recognition rate; *Sen* sensitivity, representing the rate of positive samples correctly classified by an AI model; *SFI* smartphone-based fundus images; *SD-CNN* self-defined convolution neural network method; *SDC2* syndecan 2; *SGB* stochastic gradient boosting; *SGD* stochastic gradient descent; *SMOTE* synthetic minority over-sampling; *SOM* self-organizing map; *Spec* specificity; *SSFSE* ingle-shot fast spin-echo; *SVM-RFE* support vector machine-recursive feature elimination; *TCM* traditional Chinese medicine; *TOMIM* threshold-driven optimized mutual information method; *TOPCA* threshold-driven optimized principal component analysis; *TOPSIS* technique for order of preference by similarity to ideal solution; *TPFFN* topology-preserving feed-forward network; *VIP* variable important for the projection; *VGG16* visual geometry group 16-layer network; *XGBoost* eXtreme gradient boosting.

## Results

### Baseline study characteristics

**Fig. 2:**
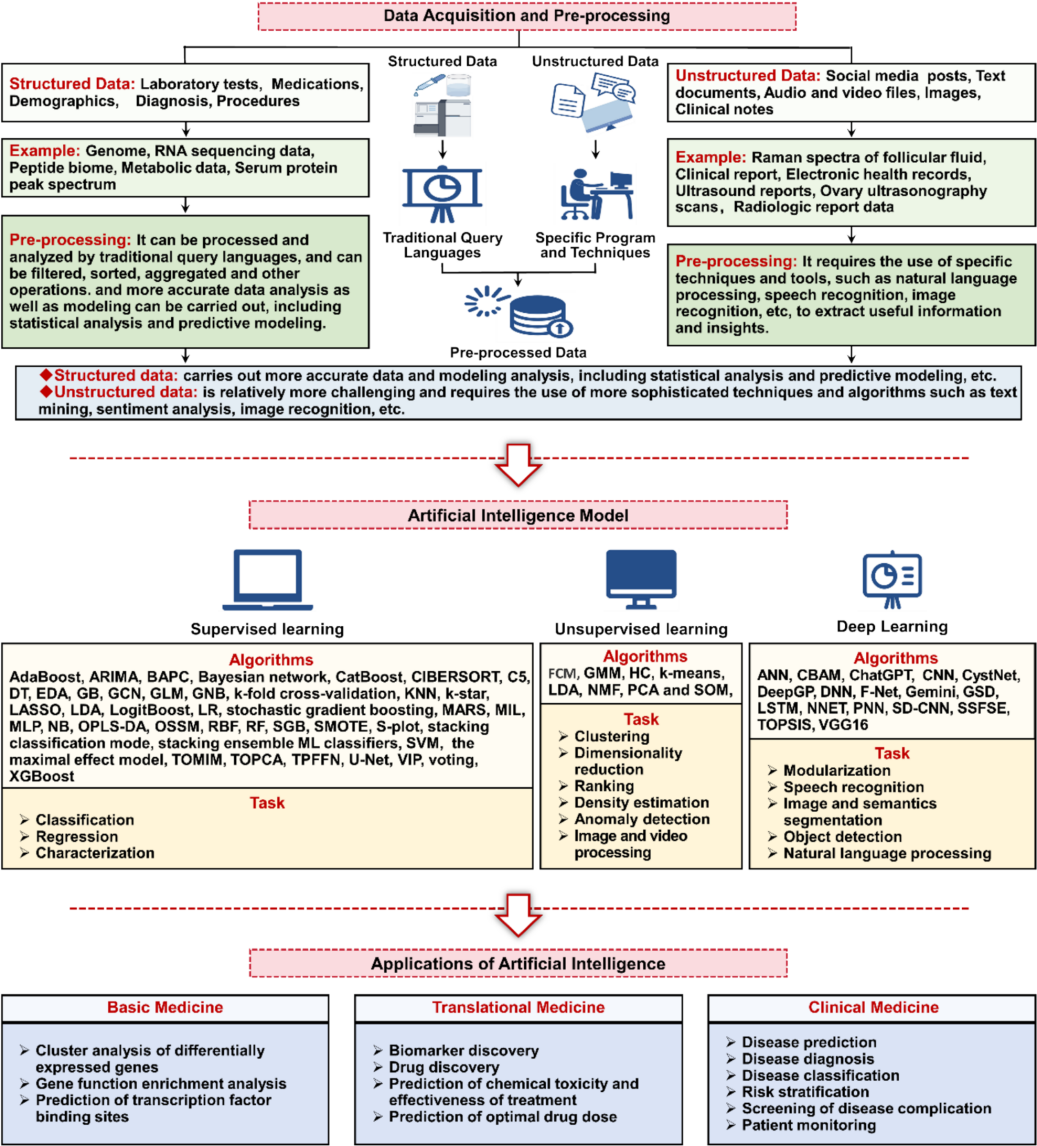
Current applications of machine intelligence in PCOS management. Common algorithms used in supervised learning include (1) artificial neural networks, such as CNN, KNN, PNN, and DNN; (2) Bayesian learning, such as NB; (3) DT, such as classification and regression tree, and supervised learning in quest; (4) ensemble methods, such as RF, and XGBoost; and (5) linear models, such as linear regression, LR, GLM, SVM. Common algorithms used in unsupervised learning include (1) clustering, such as *k*-means and GMM; and (2) dimensionality reduction, such as SOM

The application of AI in PCOS care and research has been widely explored in basic biomedical research [20], translational research [21], and clinical practice [22] (Fig. 2). According to the category of handled tasks, basic AI algorithms can be roughly divided into two types: supervised and unsupervised ML methods [23]. Via assessing the patterns in all the tagged input–output pairs such as the classification of polycystic ovary morphology (PCOM) in pelvic ultrasounds or investigating the diagnostic performance of AI on follicle counting or in another avenue, etc. [24], supervised ML methods involved collecting several"training"cases, which contain inputs (imaging, ultrasound reports, high-resolution ovary magnetic resonance imaging (MRI), etc. [25]) and the desired outputs of the task (the presence or absence of PCOS, etc.), generating the correct output for a given data type in new cases [26]. Unsupervised deduce the latent patterns in unlabeled data to find a subset of the original data (distinguishing PCOS from another disease similar to PCOS [27] or a subtype of PCOS [28, 29]). With the proposed algorithm, the outliers in the data are identified to generate low-dimensional representations of the data, or represent images and videos, and can be dealt with effectively [30].

Semi-supervised learning and reinforcement learning are also vital types of ML [31, 32]. Countless health records of PCOS administration with little supervised information (e.g., annotations of PCOM in ovary ultrasonography scans) require expensive human effort in labeling or scoring [33]. Semi-supervised learning could perform the learning tasks with considerable unlabeled data merged labeled data, utilizing unlabeled or unscored data together with only a few supervised data to optimize the performance of AI models [31]. Reinforcement learning is designed to learn an optimal strategy from data that maximizes the overall rewards [32]. Studies showed that reinforcement learning has been utilized to develop dynamic treatment regimens and provide a precise insulin dosage to react to the immediate needs of patients with diabetes, where the inspiration can be borrowed from PCOS management [34]. In addition, DL is a branch of ML based on artificial neural networks (ANN) that automatically extract abstract features from mass data (curated disease gene data, high-resolution ovary MRI, etc.) [20, 35], to make computers analyze and understand a variety of practical problems like human beings.

Generally, the rapid progress of AI methods makes novel breakthroughs and achievements for health care, whereas several potential flaws [36] such as data bias and overfitting become significant health concerns for PCOS management. The deployment of AI-driven systems for PCOS management may harbor inherent limitations, including data bias, an absence of individualized care, constrained comprehension of intricate patient histories, susceptibility to errors or misdiagnoses, and difficulties in addressing patient apprehensions and emotions. These shortcomings may culminate in suboptimal treatment regimens and patient outcomes, which will be expounded upon in the"Opportunities and challenges for both populations and clinicians"section.

### The importance of prevention strategy for PCOS

Given the general cost of care provided to reproductive-age PCOS women accounted for a relative majority of the total costs (approximately 98%), more widespread and liberal prevention for the disorder appears to be a cost-effective strategy, leading to earlier diagnosis and intervention, possibly the amelioration, and prevention of serious sequelae [37]. Thus, the prevention strategy for PCOS is structured into the following three key components based on the PCOS's progression and the underlying risk factors, referring to a hierarchical approach to the prevention and management of PCOS [38]. Primary prevention addresses risk factors such as poor diet, inadequate physical activity, and psychological stress to prevent PCOS before it starts. Secondary prevention implemented during the preclinical phase, aims to detect, diagnose, and treat PCOS early to prevent complications and disease progression. Tertiary prevention provides comprehensive management plans for diagnosed individuals, integrating medical therapy, lifestyle modifications, and psychological support to address the multifaceted nature of PCOS and enhance the quality of life. By implementing these strategies at different stages, the goal is to reduce the incidence, severity, and impact of PCOS. Ultimately, a comprehensive approach to PCOS prevention aims to improve health outcomes and enhance the well-being of affected individuals.

### AI implementation in the prediction and prevention of risk factors for PCOS

#### The rise of AI-based prediction in PCOS

PCOS has a prevalence rate of approximately 10% worldwide, accounting for 75% of ovulatory infertility, while early identification and timely treatment could avoid effectively multiple embryo transfers in abused assisted reproductive technology and fertility-sparing surgery. Thus, early screening of PCOS is essential to treat the disease and prevent its long-term complications [39, 40]. Fortunately, with the advent of AI technology, a new approach requires few manpower, material, and financial resources which is an efficient screening program conducted on a large scale for patients who are susceptible to PCOS. Al could participate in distinguishing risk factors for PCOS onset owing to method limitations and cognitive distortion when tackling large spaces of risk factors. Recently, using supervised ML, convolutional neural network (CNN), SVM, random forest (RF), and so on, plenty of studies have proposed AI applications based on the ability to accurately detect DR from clinical symptoms, genome, and ultrasound images (Table 1). In general, Al-based predicting for PCOS primarily focuses on the following five aspects.

#### Prediction based on multi-omics data

At the dawn of the twenty-first century, Zhao SY et al. [41] set up an innovative AI model incorporating SVM to facilitate the rapid and efficient identification of serum discriminatory protein profile, which facilitated the development of AI algorithms for identifying PCOS markers. Similarly, numerous polygenic, function-centric studies were conducted on the genetics of granulosa cells in follicular fluids of PCOS women, alongside the analysis of ovarian gene expression profiles sourced from the National Center for Biotechnology Information (NCBI) and Gene Expression Omnibus (GEO) databases. For instance, Coskun S et al. recognized that 10,000 international units (IU) of human chorionic gonadotropin (HCG) motivated minimal changes in the gene expression profiles of granulosa cells in follicular fluids compared with 5000 IU [42]. Next, N6-methyladenosine (m6A) regulators (YTHDF1, RBM15, and METTL14) and cuproptosis-specific molecular (COL5A1, IL18BP, SLC12A5, MDK, and RXRG) were identified successively an optimal related to the occurrence risk of PCOS, with a comparative analysis of the diverse ML as well as evaluated error rates [15, 43]. Other studies collectively illustrated that the dysregulated gene expression of IGF1, IGF2, SMAD3, CXCR1, ACP5, CEACAM3, S1PR4, and TCF7 matched PCOS disease risk by the nomogram, spurring the dysregulation of ovarian biological processes (regarding transporter activity, catalytic activity, tyrosine phosphorylation of STAT5 protein, and immune response) [44–46].

#### Establishment based on the physical examination, and laboratory

Recently, much of the proposed ML stacking ensemble techniques significantly enhanced the Acc in the case of all varieties of PCOS feature sets from the Kaggle repository, including information on demographics, biochemistry, and lifestyle (such as age, body mass index, hair growth, menstrual cycle length, follicle-stimulating hormone, endometrial thickness, and so on). These programs were applied in clinical data or electronic health records with various classifiers trained, tested, and assessed utilizing different feature sets, developing self-diagnostic prediction models of PCOS in potential patients and clinical providers, as a feasible additional technique to the error-prone and time-consuming diagnostic technique [47–53].

#### Establishment based on the history and high-risk factors

Apart from mass self-screening of the population without predisposing factors, certain high-risk populations are also the potential focus of the AI-based PCOS prediction model. Mostly, the newly developed AI predictive models were emerging toward outpatient populations who had any visit to the medical center for primary care, obstetrics, gynecology, endocrinology, general internal medicine, or family medicine, evaluating the dominant features of PCOS, based on irregular menstruation, the irregular cycle feature, hyperandrogenism, and polycystic ovarian morphology (PCOM) on ultrasound [52, 54]. Besides, an advanced AI prediction algorithm was developed to pilot-test bisphenol analogs; such endocrine-disrupting chemicals could also be a specific predictor of PCOS [55].

#### Prediction based on the PCOS vital signs

Since the diagnosis of PCOS mainly relies on ultrasonography and serology related to the women's menstrual cycle, which are strict requirements on the detection time, noninvasive and simple screening methods are required due to the patients’ low cooperation. Interestingly, it has been discovered that abnormal sex hormone metabolism and blood circulation in PCOS patients can be reflected in the appearance of body characteristics [56–58]. AI is an ideal method for offering experimental implementation of PCOS prediction from vital signs. For instance, DL algorithms such as multi-instance learning and CNN were performed in scleral images segmented from full-eye images to explore the auxiliary detection for pre-PCOS and PCOS based on the scleral changes feature, notably, which is the only existing image-based study [59]. Wang W et al. bring forth that various feature selection algorithms such as SVM, multilayer perceptron classifier, and eXtreme gradient boosting classifier with noninvasive clinical data of tongue and pulse factors can predict the existence of pre-PCOS and PCOS precisely [60].

#### Prediction of medicine efficacy and PCOS burden globally

Using an AI algorithm, predicting the effects of drug interventions on evaluating weight and hormone levels of PCOS patients became feasible, which can serve as a sensitive marker to predict treatment compliance. Several studies exerted a useful AI program such as ANN to assess the effect of carnitine or metformin supplementation related to weight and blood glucose reduction in patients with PCOS, even predicting an appropriate dosage schedule from the therapeutic index [21, 61, 62]. Their results indicated that the model developed based on the AI approach may be more time-saving and efficient and reduces the labor intensity than that based on traditional epidemiological research avenues because only the AI-based model could automate predict PCOS without extensive laboratory tests. Meanwhile, their exploration may pave the way for a reconsidering of the criteria of symptoms and signs of PCOS, contributing to better screening of a broader population included in the effective intervention or measure directed at PCOS.

Based on the commonwealth income, World Health Organization region, and the sociodemographic index, AI tools also comprehensively evaluated the global, regional, and national burden of PCOS regarding incidence, prevalence, and years lived with disability [63]. From 1990 to 2019, the annual incidence of PCOS increased significantly from 1.4 million to 2.1 million. This trend was not uniform across regions, with the Americas seeing a decrease, while countries with middle human development index levels experienced the highest increase. Besides, the highest incidence was among girls aged 10–19 years old, suggesting that the ecological validity of AI models in clinical PCOS screening trended as a promising hotspot.

### AI implementation in the diagnosis and distinction of PCOS

#### The rise of AI-based diagnosis in PCOS

Precise diagnoses are essential for formulating optimal therapeutic strategies, particularly in PCOS, where etiological factors contain clinical indexes and biochemical indicators of PCOS patients are highly heterogeneous [64]. Although a majority of research evaluated AI application in PCOS based on more than one of gynecological and endocrinological alterations, using standardized criteria such as the Rotterdam, NIH, and International PCOS criteria as reference standards fail to address the term PCOS in all the clinical manifestations observed [65]. Thus, to prevent this pattern, developing accurate diagnostic methods involved in multitudinous AI model validation from available databases and universal inspection has been considered to take priority in current research on diagnostic criteria. Al-based diagnosis for PCOS primarily focuses on the following four aspects according to data types.

#### Diagnosis based on multi-omics data

Above all, Al has been widely utilized for screening potential biomarkers of PCOS because it enables the identification of genetics and proteomic downloaded from databases such as GEO and then investigation of the protein/peptide signatures in PCOS. As early as 2007, Matharoo-Ball B et al. [66] implemented ANN analysis in Nottingham, where three optimal subsets and six biomarker ions for protein and peptide data sets were identified as predictors, respectively. Similarly, researchers compared different types of ML algorithms containing RF, SVM, ANN, and generalized linear model (GLM) to select the optimal model for prioritizing susceptible genes from the genome of PCOS (including CNTN2, CASR, CACNB3, MFAP2, BTBD9, TMOD1, PPM1B, CAMKK, MSL3, ALPK2, PAB23, RAB40C, AMPD3, SPARC, and CCR7) [20, 67–76]. Hub miRNAs of PCOS were identified based on the small RNA deep sequencing, which regulated genes with Wnt and MAPK signaling pathways, oocyte meiosis, progesterone-mediated oocyte maturation, and cell cycle functions [77]. Moreover, the ANN method outperformed all other models with the highest Acc achieved ranging from 73 to 100%, which showed the combined proteomic and genic approaches developed to be extremely robust and capable of identifying optimal biomarker subsets (containing immune-related biomarkers cAMP, S100A9, TLR8, IL6R, HDDC3, SDC2, and APOA1, and A2M) of true importance in PCOS [66, 78–80]. Next, significant differences in dopamine and linoleic acid metabolism existed in the vaginal fluid between PCOS and non-PCOS women, exhibiting its competency for identifying PCOS [81]. With unremitting efforts, driving AI to distinguish PCOS from the subtle differences of metabolic composition in serum and serum bile acid profiles, follicular fluid, and urine metabolite fingerprints (identified by high-performance liquid chromatography-tandem spectrometry) was also developing into the latest research hotspot [16, 82–88].

#### Radiology-based diagnosis

The development of automated PCOS diagnostic technologies based on ultrasound images of PCOM is opening increasing avenues for Al screening for PCOS. For the first time, it was Lehtinen JC et al. developed a new AI system for the 2-dimensional visualization of PCOS in 1997 [27]. They described a topology-preserving feed-forward network for diagnosing PCOS and compared it with the self-organizing map, which is regarded as the most widely used neural network for data visualization.

PCOM on ultrasonography is considered a core feature of PCOS, which is relevant as a diagnostic criterion for PCOS, however, there remains a lack of clarity regarding the best practices and specific ultrasonographic markers to define PCOM [89]. Considering as relatively efficient approaches, high-resolution pelvic and abdominal ultrasound images with a DL reconstruction algorithm or watershed object growing system were precise in displaying follicles, follicular fluid, and plasma identification for PCOS diagnosis, as well as improving follicle number per ovary assessment [25, 35, 90–97].

#### Diagnosis based on the history, physical examination, and laboratory

In addition to PCOM, the diagnostic criteria of PCOS cohorts focused on biomarkers identification based on the forum-posted results and extraction of electronic medical record datasets, which has gradually transformed the latest hotspot of Al screening in PCOS. The diagnostic value of ML integrated with laboratory signatures that (luteinizing hormone (LH)/follicle-stimulating hormone (FSH), progesterone, anti-Müllerian hormone (AMH), total cholesterol, triglyceride, and high-density lipoprotein cholesterol) or three clinical indicators (LH, LH/FSH, and AMH) has the potential to significantly improve the Acc of diagnosing PCOS patients [98–100]. Emanuel RHK et al. [101] conducted specific codes such as SVM, RF, and k-nearest neighbor (KNN) for capturing the levels of serum androgens, corticosteroids, progestins, estrogens, testosterone, and sex hormone-binding globulin of the steroid hormone biosynthesis pathway. Similarly, the history and physical examination were also the basis for differential diagnosis of PCOS. The nomogram model is based on 17 key features that abortion numbers, pregnancy (yes or no, Y/N), exercise (Y/N), marriage status (Y/N), weight gain (Y/N), hair growth (Y/N), cycle length (days), fast food (Y/N), weight (Kg), endometrium thickness (mm), and follicle numbers were regarded as a novel diagnostic selection to differentiate PCOS [95]. DL algorithm was exerted to estimate odds ratios of missed PCOS diagnosis via race/ethnicity, education, primary language, body mass index, insurance type, and social vulnerability index score [102]. Castro V et al. [103] and Shanmugavadivel K et al. [95] even comprehensively consider the mixed sets of radiography and laboratory.

#### PCOS distinction from other similar disorders

Due to the clinical presentation of patients with androgen excess being extremely similar in a common female condition, many national and international consensuses recommend that the diagnosis of PCOS should be systematically ruled out before the diagnosis of other special diseases associated with hyperandrogenism, signs of oligomenorrhea, and infertility [104]. Assessing those reduced sets, several ML classification algorithms were trained and tested to classify PCOS and non-PCOS patients. Bachelot, G. et al. addressed a combination of metabolomic signature and supervised ML models that may be an alternative to dynamic adrenocorticotrophic hormone testing which could distinguish non-classic 21-hydroxylase deficiency from PCOS [16].

### AI implementation in the classification and evaluation of PCOS

#### The rise of AI-based classification of PCOS

At present, based on the definition and typical characteristics of PCOS, there are three crucial diagnostic criteria for assessing PCOS, which entails confirming the presence or absence of hyperandrogenism, ovulatory dysfunction, and PCOM, wherein identifying PCOM with the assistance of ovarian ultrasonography is decisive for greater emphasis on defining the actual phenotypes of PCOS [105]. As the uncertainty of PCOS etiology and heterogeneity of PCOS clinical manifestations, the intervention continued to tamp down symptoms rather than address causes. That’s why the specific phenotypes included must be precisely recognized and documented, where AI programs may be reckoned as an exceedingly assistant for doctors to carry out repetitive diagnosis and classification routines, guiding more personalized and effective approaches to the treatment of PCOS. Al-coupled classification for PCOS chiefly focuses on the following three aspects according to data types.

#### Classification based on multi-omics data

The genetic architecture of PCOS defined by different diagnostic criteria was generally similar, suggesting that the criteria do not identify biologically distinct disease subtypes [106]. Using biochemical and genotype data from a previously published PCOS genome-wide association study and GEO database, SVM, RF, unsupervised hierarchical cluster analysis, and Gaussian mixed model (GMM) were performed to investigate the hypothesis that there were biologically reproducible phenotypic relevant subtypes of PCOS with subtype-specific genetic associations, regarding reproductive and metabolic PCOS subtypes, as well as hyperandrogenemia and non-hyperandrogenemia PCOS subtypes [29, 107]. Detailed findings performed a comprehensive metabolomics approach containing the method of pattern recognition and SVM to explore the changes in the serum protein expression profiles and the plasma phospholipid fatty acid profile [108–110]. The urine metabolic profile was used to identify the panel of urine metabolomics signature of hyperinsulinemia and insulin resistance in PCOS [111]. The AI application mentioned above helps yield promising insights into the pathogenesis and advance the diagnosis and prevention of PCOS. The plasma phospholipid fatty acid profile.

#### Radiology-based classification

Based on pelvic radiographic images and their reports, the AI approach is a handy tool for PCOS classification through direct follicle counting and PCOM evaluation. Multifunctional ML text algorithms processed information from the ultrasonic image report, along with high Acc of performance measured on the test set and inter-rater agreement between the classifiers [112, 113]. Considering the limitation of using ultrasound report text as a typical method, other researchers directly detect PCOM from the ultrasound images. Initiatively, a series of AI programs were proposed to participate in the automated classification of the ovaries based on the biomarker detected by the physician, in place of laborious manual recognition of the follicles in terms of area measurement and counting the number of follicles, which may usually lead to error-prone conclusions under fatigue, achieving a maximum efficiency of 97% outperforming all models [114]. Subsequently, the Al model based on the supervised training of CNN was executed to discern quantitative differences in endometrial histology that endometrial immune cells between cycle phases and between samples from PCOS women and non-PCOS controls [24].

#### Classification based on the physical examination and laboratory

Biochemical variables and biomarkers were also representative indicators of PCOS classification. Taking advantage of the clinical features dataset, Van der Ham K et al. proved distinct PCOS subtypes (metabolic, reproductive, and background PCOS subtypes) could also be identified to assess additional clinical variables in 2024 [115]. A fraction of the research aimed to apply the developed algorithm to select more important clinical and biochemical variables related to PCOS and to classify them into phenotypically different clusters. Silva IS et al. [22] defined the most relevant clinical and laboratory variables related to PCOS diagnosis (lipid accumulation product, abdominal circumference, age, body mass index, follicle-stimulating hormone, insulin levels, triglycerides, etc.), stratifying patients into different phenotypic clusters using ML algorithms such as RF, SVM, KNN, and the BorutaShap methods. Unexpectedly, pulse-wave parameters and traditional Chinese medicine patterns (containing the Kidney Yin Deficiency pattern, the Kidney Yang Deficiency pattern, the Phlegm-Dampness pattern, the Blood Stasis pattern, and the Qi Stagnation pattern) in PCOS patients were also successively classified under ML methods [116, 117].

### AI implementation in screening complications and educating patients

#### The rise of AI-based screening in PCOS complications

Heterogeneous by nature, PCOS is defined by a combination of signs and symptoms in the absence of other specific diagnoses, although the etiology of which remains mysterious, the diagnosis and intervention of PCOS and its complications are not complicated [118]. Wherein all complications, extensive reports show that it is extremely important to investigate the leading effects in damaged pregnancy and offspring development of PCOS patients [119, 120]. Fortunately, one of the essential functions of AI-mediated methodology is predicting the prevalence likelihood of complications, as well as identifying potential shared diagnostic genes between spontaneous complications and their risk factors, which allows healthcare professionals to further facilitate better disease systematic management [34]. Notably, almost all of the studies in this domain were published in 2024, revealing the growing emphasis on optimizing the practical guidance of AI algorithms in physician–patient-supported synergetic PCOS management involving clinical decision development and patient education. Hence, we performed a systematic review of AI interventions in PCOS complication prediction from the following three aspects.

#### Prediction based on multi-omics data

To date, omics data for ML algorithms of PCOS complications assessment in the existing literature are mainly gene and protein information obtained from clinical samples and biological information databases (the GEO and Gene Cards database), as well as biological components of follicular fluid analyzed by Raman spectra. Among all the complications, plentiful reports showed a top priority in evaluating their predictive value for oocyte development potential and damaged obstetrical outcomes that recurrent implantation failure and recurrent spontaneous abortion in PCOS patients; thus, the priority jumped to early prediction of embryonic developmental potential and adverse clinical pregnancy [119, 121, 122]. Combined with the available data on basic sex hormones, embryonic morphology, the protein composition of endometrial, the follicular microenvironment, and the negative emotion of PCOS, the regulation of GLIPR1, FAM166B, TIA1, MAMLD1, and COL5A1 was evaluated from transcriptomics and metabolomics data, predicting pregnancy outcomes under the RF, SVM, least absolute shrinkage and selection operator (LASSO) algorithms, and so on [123–127]. Next, RF was used to evaluate the ability of ferroptosis-related genes (ATF3, BNIP3, DDIT4, LPIN1, NOS2, NQO1, SLC2A1, and SLC2A6) in granule cells to predict damage oocyte quality of PCOS women [128]. Apart from the pathological disorder associated with female fertility preservation, PCOS patients with chronic low-level inflammation, dyslipidemia, insulin resistance, and hyperandrogenism were also susceptible to hypertension, atherosclerosis, and other cardiovascular diseases with awful morbidity and mortality [129]. The identification of virulence genes for PCOS-associated endometrial carcinoma was also analyzed by AI algorithms LASSO, SVM, and the topological matrix, indicating both NAA15 linked to sedentary behavior and ENPP2 in hormonal response within PCOS were the novel biomarker for endometrial carcinoma [130, 131]. MMP9, DAPK1, and P2RY13 tracked by flourishing AI nomogram (LASSO, RF, and SVM) were negatively related with Macrophages M1, translating as PCOS-mediated hub genes in atherosclerosis, with the latent pathogenesis lies in inflammatory response and immunity [132, 133]. The mitochondrial functional gene NPAS2 was the hub gene. Using LASSO and SVM, Liang H et al. [134] raised the mitochondrial functional gene NPAS2 as the early diagnosis biomarker of major depressive disorder in PCOS women. The current studies assuredly spark new inspiration in diagnosing and treating PCOS complications.

#### Predicting based on the physical examination and laboratory

In view that PCOS treatments are merely symptomatic, the focus has shifted to embryo quality and pregnancy outcomes of patients with PCOS [135], and the performance of AI tools would be described according to whether the observation of outcome indicators was associated with female fertility preservation. Thus, a majority of articles provided a comparative analysis according to oocytes, sperm, embryo quality, and couples'characteristics to classify successful deliveries, providing model explanations to ensure efficiency, effectiveness, and trust of the developed model in the early detection and treatment of PCOS with the possibility of long-term complications. Such usage in practice was easy to find, combined with theoretical framework and mathematical validation [125, 136–139].

In addition, specific factors may be explicit culprit risk factors too. Screening an integration of the dynamic Bayesian network system in clinical practice, researchers aimed to reveal the likelihood of pregnancy under higher androstenedione and hyperhomocysteinemia-induced recurrent implantation failure in PCOS patients, based on endometrial histology features and calculating the areas occupied by epithelial and stromal cells [140]. An unsupervised ML algorithm was driven to discern that significantly higher luteinizing hormone, free androgen index, and androstenedione levels were linked to a higher metabolic syndrome score caused by PCOS [28]. Similarly, mitochondrial DNA copy numbers were defined as negatively correlated with waist-to-hip ratio and triglycerides, positively related to high-density lipoprotein cholesterol in PCOS [141]. Besides, the features of PCOS and diabetes were deeply analyzed and remarked as a positive correlation of 95% utilizing exploratory data analysis [142]. Interestingly, the tendencies of mental health issues concerning anxiety and major depressive disorder were also deemed as an outcome indicator derived from PCOS-related research [143, 144]. Eventually, ML exhibited the highest Acc in predicting discordance, showing that PCOS women with less body fat mass, skeletal muscle mass, and ultra-low-dose pills had a lower risk of acquiring future atherosclerotic cardiovascular disease [145, 146]. In general, AI systems demonstrate exceptional proficiency in distilling intricate patterns within virulence genes or risk factors, meticulously ascribing these partial attributes to an ordered framework that aligns with global structural characteristics. Through this refined process, the systems are trained to execute comparative analyses and screenings, ultimately surpassing the efficacy of traditional clinical research endeavors.

#### Efficacy evaluation of AI on PCOS management

Facing complicated conditions that may profoundly impact daily life, PCOS patients were more inclined to address their concerns online for nutritional recommendations and necessary lifestyle management, underscoring the reliability of accessible digital medical resources [147, 148]. Thus, the extent, content, and engagement of PCOS-related information across social media platforms such as TikTok, Instagram, and Reddit were assessed under linear Discriminant and logistic regression analysis [149]. Additionally, open AI tools such as Chat Generative Pretrained Transformer (ChatGPT) and Gemini built neural networks with training data, differentiating for their high quality in diverse text or voice prompts for PCOS-related infertility management [150]. In terms of quality, Acc, and readability, however, general advice from AI applications was still inferior to personalized medical guidance from healthcare professionals, which were given a slightly lower average score based on subjective scales (such as DISCERN, global quality scale, and Likert) [151, 152].

## Summary

**Fig. 3:**
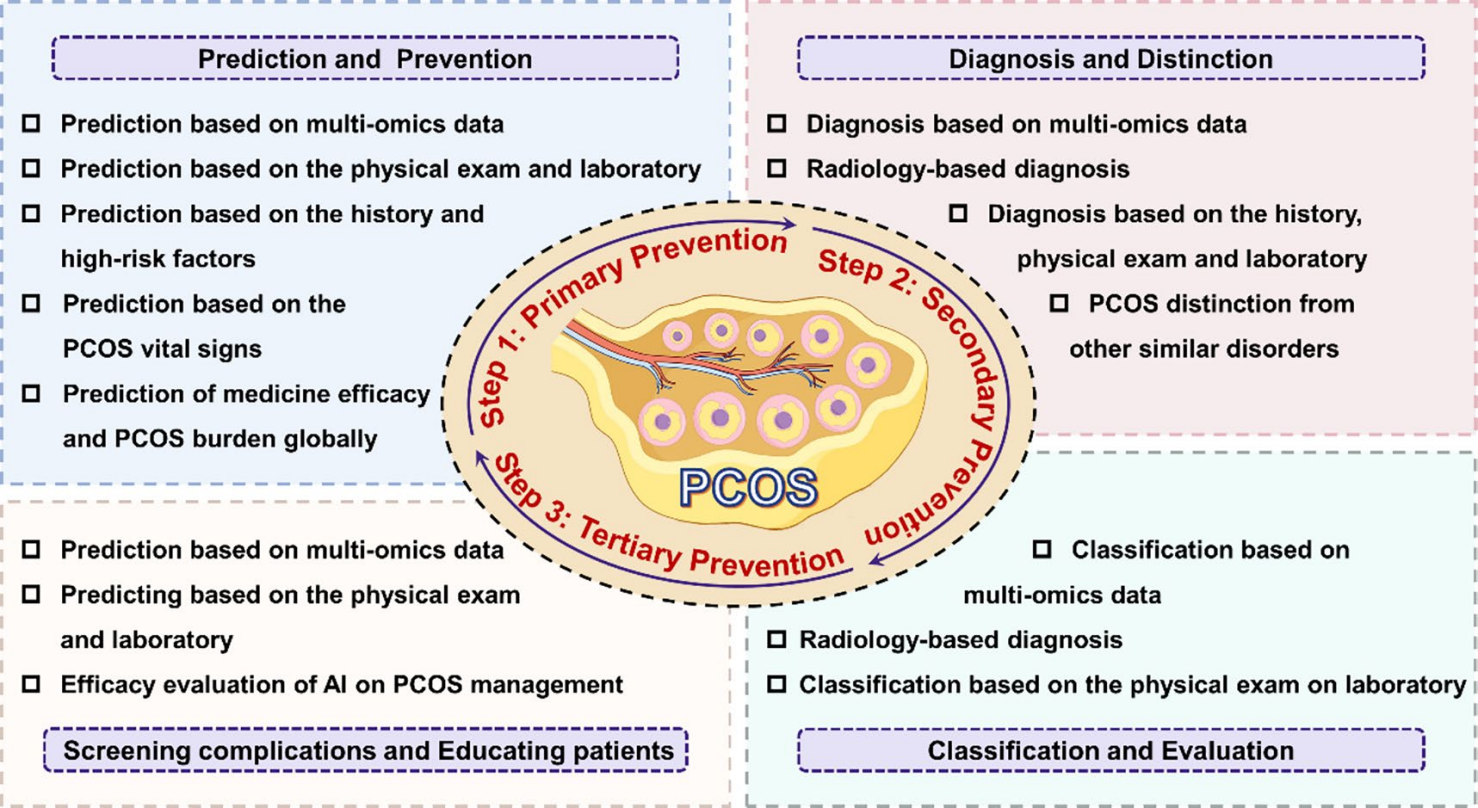
Overview AI implementations in PCOS management based on prevention strategies

With time and technological advances, the focus has shifted to better life quality, and the increasing morbidity of infertility has become a global public health challenge in the twenty-first century. As the most common cause of ovulatory infertility, the high heterogeneity of PCOS clinical manifestations fueled the difficulties in strategies formation of PCOS diagnosis, treatment, and management. The newest emergence of AI may help address these obstacles and alleviate the disease burden of PCOS via computing hardware advances in future. Early studies have revealed that AI application of PCOS management permeated every stage of the typical tertiary prevention strategy including prediction and prevention, diagnosis and distinction, classification and evaluation, as well as complications screening and patient education of PCOS. The harnessing of AI with clinical practice care extremely catalyzed the development of PCOS care in the direction of precision, penetration, prediction, and personalization (Fig. 3).

### Validity of AI systems

#### Data issues in developing and integrating Al systems

**Fig. 4:**
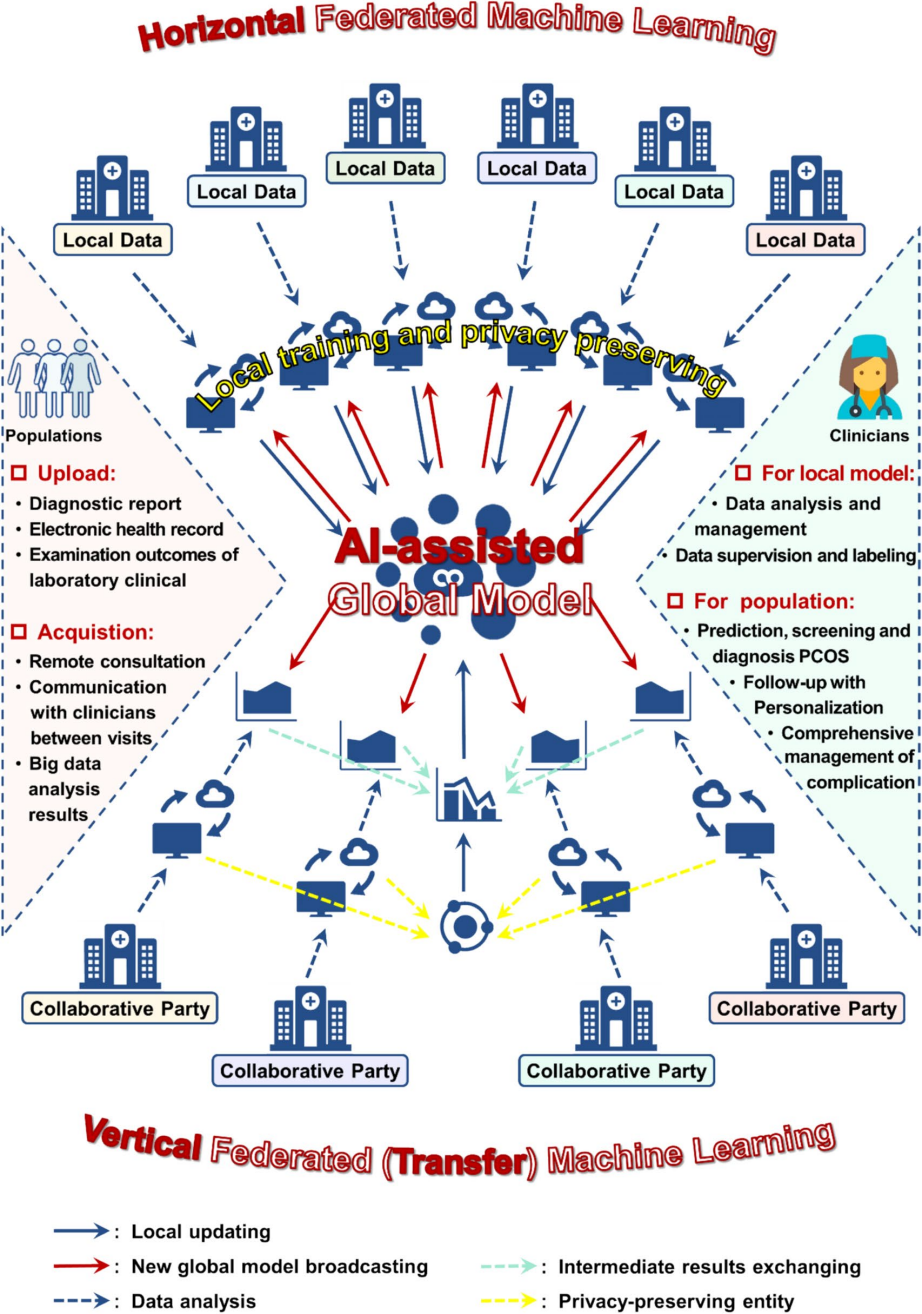
The framework of federated learning. According to the data partition, it’s mainly classified into horizontal and vertical federation learning models. The horizontal federation learning model applies to the condition where different users in the same domain have similar characteristics. Local hospitals got copies of the current global model from a federated server to train on their datasets, sending the model updates back to the federated server with a certain number of iterations, keeping their datasets in their secure infrastructure. The federated server aggregates the contributions from these hospitals. Then the updated global model is shared with the local hospitals and they can continue local training. The most typical case is that a local hospital learns the medical experience of users from the diagnosis and treatment information of different users, providing relevant information on disease management for users according to the information with self-optimization. Vertical federation learning is suitable for situations where users in different domains have common data (data characteristics are not consistent). The training steps are as follows: 1) The coordinator is responsible for distributing the public key so that only coordinator can decrypt it; 2) participants carry out homomorphic encryption and interaction of the aligned samples, as well as calculate their respective gradient and loss values respectively; 3) after participants are calculated, they are sent to privacy-preserving entity (a mask or noise will be added at this time to avoid leaking); 4) privacy-preserving entity decrypts it, sending it back to participants which unmask and update their models. The main advantage of federated learning is that it establishes a global model without directly sharing datasets, preserving patient privacy across sites

Considering the complex diagnostic criteria spanning clinical, biochemical, and radiological domains, delayed detection, high morbidity, and significant annual healthcare expenses, PCOS is a potent target for AI-based tools. Thirty percent of women delay diagnosis for more than two years implying PCOS is on behalf of an ideal setting for future AI-mediated approaches [147]. Robust AI systems certainly are going to spring up in future as computing hardware and technology advance, which promise new forms of PCOS care to reduce the huge burden on families and caregivers and high costs for medicine and surgical interventions. Large datasets are required to fuel the optimization of a blooming AI system where data sharing might be a preferred solution, while it results in ethical and legal challenges generally. As federated learning offers more privacy than a server model, it may be a valuable option for designing sensitive data collection methods, and this approach will notably accelerate the introduction of precision medicine [153, 154]. This strategy could handle the issue that data exist in different institutions, eliminating obstructions to data sharing and circumventing the problem of patient privacy. Hence, we put forward the construction of an AI-assisted digital healthcare ecosystem for PCOS management mainly characterized by horizontal and vertical federated ML (Fig. 4).

#### The opportunities and challenges for both population and clinicians

As the major participant involved in the AI ecosystem, there is no doubt that the population with/without PCOS and clinicians play an important role in AI-based operations. Open datasets, efficient labeling techniques, code-free automated ML, and cloud-based platforms for deployment are resources that enable clinicians and individuals with scarce resources to drive their own Al adoption [155].

AI projects boomed up a new scientific and technological revolution, and its successful development rapidly spreads the popularity of users. On the one hand, individuals can not only contribute to the optimization of the federated system via uploading diagnostic reports, electronic health records, examination outcomes of laboratory clinical, etc., but also acquisition from self-help actions such as remote consultation, communication with clinicians through visits, and dig data analysis results to implement PCOS screening in high-risk populations or administration [156]. On the other hand, for the local model, clinicians could perform data analysis and management, data supervision, and labeling. For the population, clinicians could predict, screen, and diagnose PCOS follow-up with personalization comprehensive management of complications [157]. Thus, mature federated ML assists medical practitioners and individuals jointly devoted to health education and disease prevention, multiple treatment modalities concerning medical nutrition, physical and drug therapy, basic management of PCOS, and so on.

Al's increasing popularization is stepping into a period where clinicians and patients stand to benefit tremendously, whereas the current plight of PCOS, even in the field of reproductive medicine, lies in the democratization of Al's practice, which couldn't be neglected as well. To begin with, regulated access datasets and diverse formats of large datasets in high-quality public datasets such as compressed zip folders, MATLAB, and various raw images may result in long download times or failed downloads, which fewer clinicians are well versed with, leading to a barrier for greater visibility of available to data [158]. Furthermore, almost none of the ML models on the path to the real-world clinic, stemming from the crucial dilemma that only a fraction of algorithms has received the approval of medical ethics committees and the government, still deter the widespread use of AI for individuals [155]. All in all, the solution of data sharing, annotation, and other related problems will facilitate the development of strong AI systems.

The constraints of AI-driven PCOS management were also assessed exhaustively by the Web of Science and CiteSpace bibliometric software.‌ Key research foci and developmental trends emerged through directional research and keyword clustering. ‌Bibliometric analysis identified major areas as Endocrinology & Metabolism (27%), Obstetrics & Gynecology (20%), Reproductive Biology (15%), Science & Technology (13%), and Biochemistry & Molecular Biology (11%). However, Radiology, Nuclear Medical Imaging, Health Care Sciences, Cell Biology, and Integrative Complementary Medicine remain underexplored yet hold practical value. Research diversity is limited, often focusing on single diseases or diagnostic mechanisms, potentially hindering holistic AI-based diagnostics. Keyword cluster analysis revealed limited integration between PCOS and emerging fields like deep learning, artificial intelligence, and bioinformatics. Despite the exploratory potential, challenges persist in standardizing subjective image analysis, processing detailed imaging data, and managing multimodal image analysis, cloud storage, data sharing, and interconnected medical records (Supplementary file 2, Fig. S1A). Analysis of the collaboration network reveals a notable absence of extensive, deep international partnerships in this research domain. Chinese AI research predominantly relies on single-center, small-sample studies, which may raise a crisis in the credibility of findings. Contributions to integrating PCOS clinical management with AI technology are largely confined to China and America, with other nations trailing in both output and quality, highlighting the influence of national development on AI and medical technology fusion. Thus, while this paper offers a thorough examination of AI-driven PCOS management, it acknowledges limitations in addressing broader research scopes and disciplines (Supplementary file 2, Fig. S1B).

#### Exploration for horizontal federated ML in PCOS management

Horizontal federated ML is an approach that data sets with more overlapping user features but fewer overlapping users, splits according to horizontal (that means user dimension), and extracts the part of the data with the same user features but not the same users for training [159]. Given PCOS management, as federated ML serves for rare cancer boundary detection and therapeutic effect evaluation for triple-negative breast cancer [160, 161], even if several institutions in a region with their patients diagnosed with PCOS will have similar businesses cooperate, and the characteristics that need to be analyzed (PCOM, the incidence of PCOS, etc.), local hospitals could still share self-renewal global model without directly sharing datasets while maintaining their privacy. Initially, each participant downloads the latest model from a central aggregator and then computes the model parameters (imaging, electronic case report, etc.) locally (aka"gradients"), subsequently sending the encrypted gradient information to the aggregator. Then the aggregator performs security aggregation operations (the common algorithm is FedAvg) for updating the AI model comprehensively, which goes along with the distribution to all local hospitals. Eventually, participants escalate their initial models. After several iterations, the Acc (the"loss function") reaches a set value, indicating the model completed the global model, where diverse organizations could collaborate on the development and optimization of AI methods aimed at practical issues such as the estimation of whether the irregular cycle feature generates PCOS.

#### Exploration for vertical federated ML in PCOS management

Vertical federated ML is an approach that data sets with more overlapping users but less overlapping user features, splits according to vertical (that means feature dimension), and extracts the part of the data with the same user but not the same users’ characteristics for training [162]. Similar to the applications of vertical federated ML in disease healthcare, cancer prognosis prediction, and other industries where data privacy is a major concern [163], in terms of PCOS management, for example, there are partially overlapped patients diagnosed PCOS or not in health clinics and reproductive hospital in a region. The health clinics’ datasets are the user's diagnosis and treatment information; however, the reproductive hospital’ datasets are the user's fertility preservation information. They could still jointly develop a risk control model without disclosing the data to each other via aligning the same user sample with different participants (encrypted entity alignment), accompanied by encrypted model training on these samples (the right part in the figure below). By vertical federated ML, diverse organizations could collaborate on the development and optimization of AI methods aimed at practical issues such as the evaluation of PCOS predictive value for oocyte development potential and clinical pregnancy.

#### Exploration for transfer federated ML in PCOS management

Transfer federated ML is a special category of federated ML, that is, the user samples and characteristics are both partial overlaps [164]. As the success of transfer federated ML in electroencephalographic signal classification [165], the architecture in the brain–computer interfaces with higher classification Acc could be learned for AI-assisted PCOS management. Transfer federated ML seamlessly integrates into the management of PCOS prevention by harnessing data from disparate sources, all while maintaining stringent privacy and security measures. Through a tiered methodology, transfer federated ML enables the refinement of model training using heterogeneous datasets, thereby enhancing the precision of PCOS risk assessments. This collaborative framework facilitates the implementation of tailored prevention strategies across primary, secondary, and tertiary levels, effectively addressing the intricate nature of the condition and fostering personalized patient care. Transfer federated ML achieves these objectives by aggregating data from multiple institutions in a privacy-preserving manner, bolstering model performance through joint learning processes, and enabling the formulation of customized intervention strategies at various stages of prevention.

## Conclusion

Al in reproductive medicine has made significant breakthroughs and achievements over the past few years. A majority of studies have verified that Al's performance is parallel to and even better than that of clinicians in many PCOS diagnostic and predictive tasks. In conjunction with multi-omics bioinformatics, clinical characteristic data, and imaging information, AI tools emphasized its potential role in development of comprehensive management strategy for PCOS, which stratifies women with potential or confirmed PCOS into distinct cohorts, enabling personalized interventions and tiered management of reproductive and metabolic complications. AI exhibits notable ecological validity in the clinical screening of PCOS, achieved through the efficient analysis of patient data and the customization of screening algorithms to accommodate diverse patient demographics. This advanced technology has demonstrated considerable proficiency in detecting PCOS, thereby tackling the intricate challenges posed by the condition's heterogeneous and multifaceted nature. However, much research remains to be done before AI is broadly utilized in clinical PCOS administration. The availability and effectiveness of AI methods are poorly investigated, appealing to paying more attention in future studies. Despite not being completely mature yet, we still prospect Al will play a crucial role in future of PCOS management even reproductive medicine, making healthcare a huge efficiency, interpretability and generalizability, especially in regions coupled with huge burden on families and caregivers, as well as high costs for medicine and surgical interventions rendered by lacking reproductive medicine specialist.

## Supplementary Information

Below is the link to the electronic supplementary material.

Supplementary file1 (DOCX 451 KB)

## Data Availability

All data generated or analyzed during this study are included in this published article, with a full search strategy in supplementary information files.

## Abbreviations

PCOS: Polycystic ovary syndrome
AI: Artificial intelligence
ML: Machine learning
DL: Deep learning
SVM: Support vector machine
AUC: Area under the curve
PCOM: Polycystic ovary morphology
MRI: Magnetic resonance imaging
CNN: Convolutional neural network
RF: Random forest
NCBI: National Center for Biotechnology Information
GEO: Gene expression omnibus
m6A: N6-methyladenosine
ANN: Artificial neural network
GLM: Generalized linear model
Acc: Accuracy
RBF: Radial basis function
GMM: Gaussian mixed model
KNN: K-nearest neighbor
Y/N: Yes or no
LASSO: Least absolute shrinkage and selection operator
TOPSIS: Technique for order of preference by similarity to ideal solution
