# Supplementary material for: Artificial intelligence in polycystic ovarian syndrome management: past, present, and future
Source: Radiol Med. 2025 Jun 23;130(9):1409–41. doi: 10.1007/s11547-025-02032-9 (PMC12454626; doi:10.1007/s11547-025-02032-9)
Supplement: Supplementary file 1 — Supplementary file1 (DOCX 451 KB) [file 11547_2025_2032_MOESM1_ESM.docx]

**
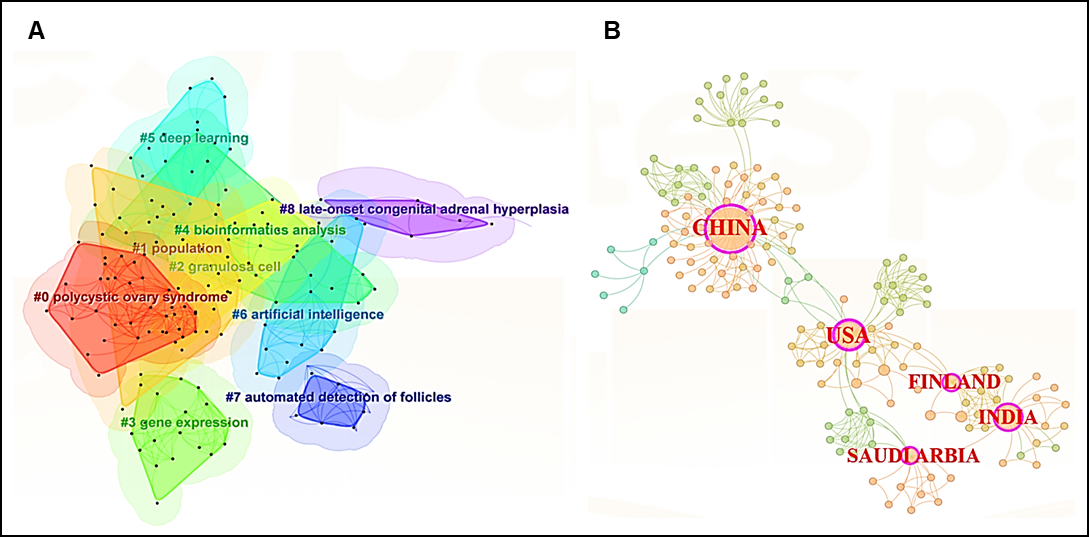
**

**Figure S1 Bibliometrics of AI-based PCOS management. A.** Clustering map of keywords. The smaller cluster label number means more keywords, and the links and overlapping areas of the cluster represent disciplinary cooperation, crossover, and integration. **B.** Country and author co-occurrence network map. The size of the country circle represents the number of publications and the pink circle in the outer layer of the country represents the centrality, with higher centrality representing higher contributions. the connecting lines represent author collaborations, the color represents the duration of the study.
